# Regulation of NLRP3 Inflammasome by Phosphorylation

**DOI:** 10.3389/fimmu.2018.02305

**Published:** 2018-10-08

**Authors:** Nan Song, Tao Li

**Affiliations:** ^1^Beijing Tropical Medicine Research Institute, Beijing Friendship Hospital, Capital Medical University, Beijing, China; ^2^State Key Laboratory of Proteomics, Beijing Institute of Basic Medical Sciences, National Center of Biomedical Analysis, Beijing, China; ^3^State Key Laboratory of Toxicology and Medical Countermeasures, Beijing Institute of Pharmacology and Toxicology, Beijing, China

**Keywords:** innate immunity, inflammasome, NLRP3, phosphorylation, kinase, phosphatase

## Abstract

The cytosolic pattern recognition receptor (PRR) NOD-like receptor family, pyrin domain containing 3 (NLRP3) senses a wide range of pathogen-associated molecular patterns (PAMPs) and damage-associated molecular patterns (DAMPs). Upon activation, NLRP3 triggers the assembly of inflammasome via the self-oligomerization and the recruitment of apoptosis-associated speck-like protein containing a caspase-recruitment domain (ASC) and pro-caspase-1, facilitating the robust immune responses including the secretion of proinflammatory cytokines and pyroptosis. The NLRP3 inflammasome must be well orchestrated to prevent the aberrant activations under physiological and pathological conditions, because uncontrolled activation of NLRP3 inflammasome is one of the major causes of a variety of autoimmune diseases and metabolic disorders. Therefore, understanding the molecular mechanisms for controlling NLRP3 inflammasome activation may provide novel strategies for the treatment of NLRP3-related diseases. Although NLRP3 inflammasome can be regulated at the transcriptional level, the post-translational modification (PTM) of NLRP3 as well as other inflammasome components has also been showed to be critical for the regulation of its activation. Several kinases and phosphatases have been shown to control NLRP3 inflammasome activation in response to either exogenous pathogen infections or endogenous molecules, such as bile acids. In this review, we summarize our current knowledge of phosphorylation patterns and their functional role in the regulation of NLRP3 inflammasome, and suggest interesting areas for future research.

## Introduction

Inflammasomes are large multi-protein complexes that mediate the immune responses against pathogen infection and tissue damage (1, 2). Canonical inflammasome is mainly composed of the initiator protein such as pattern recognition receptors (PRRs), the adaptor protein called ASC (Apoptosis-associated speck-like protein containing a CARD), and the effector protein, pro-caspase-1 (2–4). In response to the disturbance of tissue homeostasis, PRRs, such as NLRs and ALRs, undergo conformational change and nucleate the oligomerization of monomeric PRR proteins (4). Once the core PRR oligomer is formed, ASC can then be recruited and interact with the PRR oligomer through their pyrin domains (PYD) (5). As a platform, the clustered ASC can recruit pro-caspase-1 to the PRR-ASC complex via their caspase-recruitment domain (CARD) (6). This recruitment converts dormant pro-caspase-1 into active caspase-1 by the proximity-mediated cleavage (7). Active caspase-1 then promotes the proteolytic cleavage of inflammatory cytokines including pro-interleukin-1β (pro-IL-1β) and pro-IL-18, and also the Gasdermin D (GSDMD) (8, 9). The pore-forming activity of GSDMD can then trigger pyroptosis, facilitating the secretion of a serious of cytokines including cleaved IL-1β, IL-18, and IL-1α (10, 11).

So far, five different types of inflammasomes have been identified, distinguished by their core PRRs, which are NLRP1, NLRP3, NLRC4, Pyrin, and Absent in Melanoma 2 (AIM2) (3, 12–18). Although sharing highly resembled downstream pathways, each inflammasome responds to different kind of stimuli (19). Particularly, NLRP3 inflammasome senses a wide array of stimuli such as ATP, toxins, RNA viruses, and crystals (20–24). Moreover, aberrant activation of NLRP3 caused by mutations has been implicated in the pathogenesis of Cryopyrin-associated periodic syndrome (CAPS) and other inflammatory diseases (25–27). Therefore, the molecular mechanism of NLRP3 inflammasome activation has been extensively investigated.

To data, a two-step model of NLRP3 activation has been well documented, which is triggered by two sequential signals (3, 28–30). Unlike other PRRs, the basal level of NLRP3 in macrophage is quite low (30). Before activation, NLRP3 needs to be “primed” by Toll-like receptor (TLR) agonists such as lipopolysaccharide (LPS). Activation of TLR signaling not only transcriptionally upregulates NLRP3 expression, but also post-transcriptionally activates NLRP3 by phosphorylation and deubiquitination. This step has been referred to as “priming.” The second step, defined as “activation,” can be induced by several potent stimuli such as pore-forming toxins, leading to the oligomerization of NLRP3 and the subsequent assembly of inflammasome. Therefore the activation of NLRP3 inflammasome is tightly regulated by different mechanisms mediated by interacting proteins and/or modifications (31–34). Of note, growing evidence highlights that phosphorylation, one of the most abundant PTM, serves as one of the predominant signals controlling inflammasome activation (35). Several inflammasome components, such as the core PRRs like NLRP3, NLRC4, Pyrin, the adaptor ASC, and the effector caspase-1, are all reported to be regulated by phosphorylation (32, 35–44).

**Figure 1:**
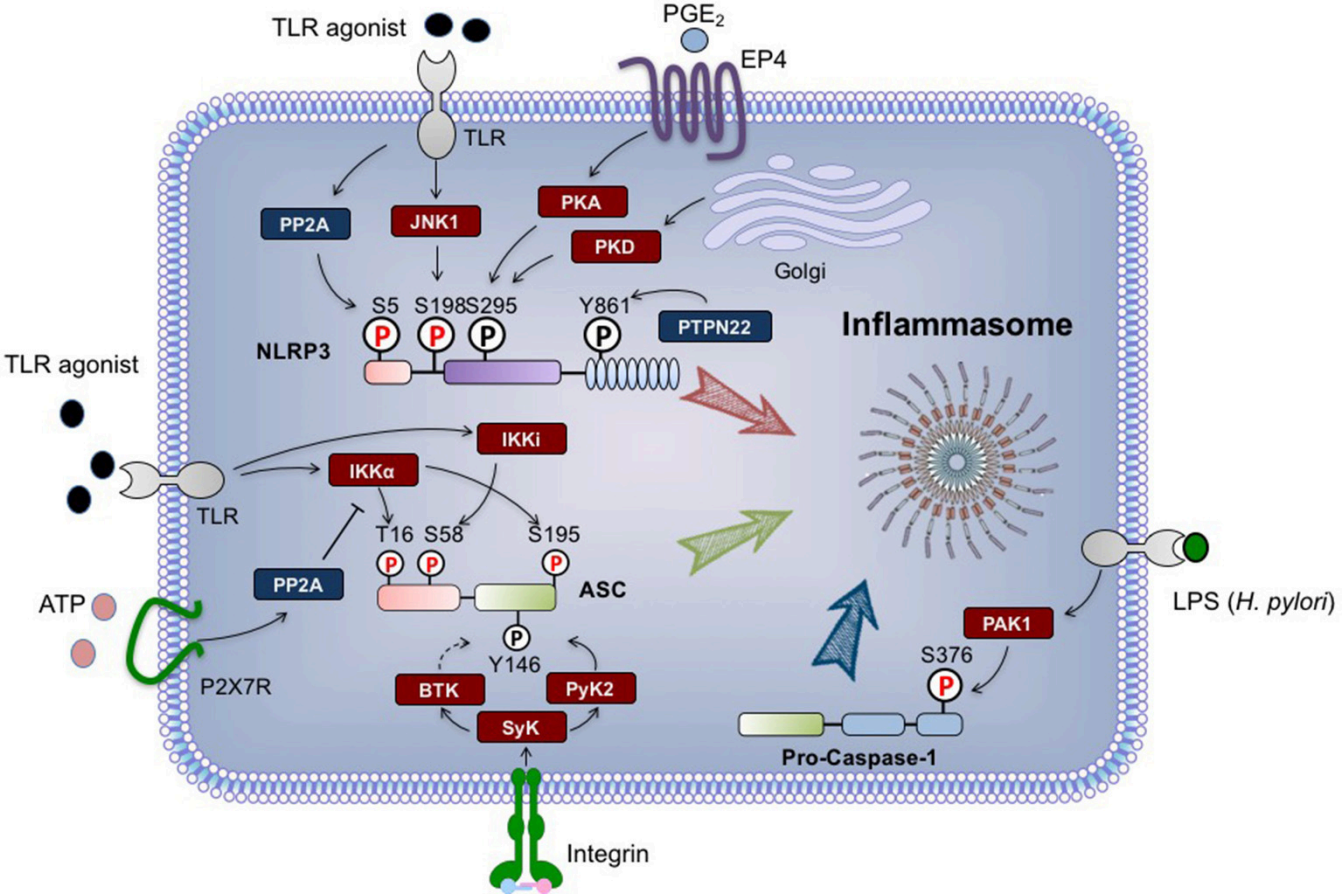
Regulation of NLRP3 inflammasome by phosphorylation. The phosphorylation modifications of NLRP3 (Nod-like receptor protein 3), ASC and pro-caspase-1 are indicated. The phosphorylations regulated by signal 1 are highlighted in red, while signal 2-regulated phosphorylations are in black. Briefly, during priming process, NLRP3 can be dephosphorylated at S5 by PP2A, and phosphorylated at S198 by JNK1. ASC is phosphorylated at S16 and S193 by IKKα, and phosphorylated at S58 by IKKi. *H. pylori*-derived LPS can activate PAK1, which in turn phosphorylates pro-caspase-1 at S376. Upon signal 2 stimulation, PTPN22 dephosphorylates NLRP3 at Y861, while ASC undergoes phosphorylation at Y146. Both of these events are required for inflammasome assembly. NLRP3 S295 phosphorylation triggered by PKD promotes the release of NLRP3 from mitochondria-associated membranes, and also negatively regulates NLRP3 activation in a PKA-dependent manner. All residue numbers refer to the human proteins. ASC, apoptosis-associated speck-like protein containing a CARD; BTK, Bruton‘s tyrosine kinase; EP4, E-prostanoid 4; IKK, IκB kinase; JNK1, c-Jun N-terminal kinase 1; P, phosphate; PAK1, p21-activated kinase 1; PGE_2_, prostaglandin E_2_; PKA, protein kinase A; PKD, protein kinase D; PP2A, protein phosphatase2A; PTPN22, protein tyrosine phosphatase, non-receptor type 22; Pyk2, proline-rich tyrosine kinase 2; Syk, Spleen tyrosine kinase; TLR, Toll-like receptor.

Here, we summarize the progress in understanding the role that phosphorylation plays in the regulation of NLRP3 inflammasome activation, including how phosphorylation can affect the activity of NLRP3 and other inflammasome components in both priming and activation steps (Figure 1).

## Phosphorylation in the priming step

Considering the limited expression level of NLRP3 in quiescent macrophages, the priming step has initially been demonstrated as a prerequisite for NLRP3 transcription (30). Nuclear factor-κB (NF-κB) can be activated by TLR signaling, and upregulate the expression of NLRP3, as well as pro-IL-1β. However, emerging evidence indicates that a rapid priming within 30 min is sufficient for the activation of NLRP3 inflammasome, suggesting that rather than transcription, another layer of regulation may be more essential for the priming of NLRP3 inflammasome (31, 45–47). A couple of studies showed that the deubiquitination of NLRP3, which occurs in both steps of NLRP3 inflammasome activation, may be responsible for the rapid priming of NLRP3 (31, 34). By screening the library of deubiquitinase, (48) demonstrated that BRCC3 functions as a key regulator for NLRP3 activation by promoting its deubiquitination (34). Moreover, one targeted chemical screening demonstrated that inhibitors that target IKK or its downstream pathway shows little effects on LPS/ATP-induced caspase-1 activation in THP-1 cells, while the inhibitors acting upstream of the IκB kinase (IKK) complex indeed blocks both NF-κB and inflammasome activation (47). This study suggested that TGF-β-activated kinase 1 (TAK1), the mitogen-activated protein kinase kinase kinase (MAPKKK), or other TLR signaling-related kinase(s), may phosphorylate NLRP3 or other inflammasome components, which serves as a rapid but essential step for NLRP3 priming.

## Phosphorylation by IKKs

In line with this hypothesis, both IKKα and IKKi have been described to play an important role in ASC phosphorylation (Table 1). Martin et al. demonstrated that the dual phosphorylation of Ser16 and Ser193 of ASC by IKKα negatively regulates the binding of ASC to NLRP3 by sequestering ASC in the nucleus (38). In contrast, IKKi/IKKε can bind with ASC and phosphorylate Ser58 site during LPS-induced priming, which facilitates the translocation of ASC from the nucleus to the perinuclear region. Although the phosphorylation of ASC may be required for all the ASC-containing inflammasome, the serine/threonine phosphatase PP2A, which dephosphorylates IKKα, specifically regulates the activation of NLRP3 inflammasome, but not AIM2 inflammasome, suggesting that ASC phosphorylation may not be the only mechanism of LPS priming (38). Moreover, not all the inflammasomes need to be primed, whereas some of them, such as AIM2 inflammasome, indeed need ASC as the adaptor to recruit caspase-1 (17). How ASC is activated and translocated during the activation of these inflammasomes need to be clarified.

**Table 1:** Regulation of NLRP3 inflammasome activation by phosphorylation.

**Phase**	**Protein**	**Site**	**Kinsae**	**Phosphatase**	**Effect**	**Mouse model**	**References**
Priming	NLRP3	Ser198 (human)	JNK1		Positive	NLRP3-S194A KI, JNK1 KO	(32)
		Ser194 (mouse)					
	ASC	Ser16 (mouse)	IKKα		Negative	IKKα-K44A KI, IKKα-AA KI	(38)
		Ser193 (mouse)					
		Ser58(mouse)	IKKi		Positive	IKKi KO	(38)
	Caspase-1	Ser376 (human)	PAK1		Positive		(36)
Activation	NLRP3	Ser5 (human)		PP2A	Negative		(42)
		Ser3 (mouse)					
		Ser295 (human)	PKA, PKD		Positive or negative	PKD1 KO, PDK3 KO	(39–41)
		Ser291 (mouse)					
		Tyr861 (human)		PTPN22	Negative	PTPN22 KO	(43)
		Tyr859 (mouse)					
	ASC	Tyr146 (human)	Syk/JNK1/JNK2, PyK2		Positive	Syk KO, JNK1 KO, JNK2 KO	(37, 44)
		Tyr144 (mouse)					

## Phosphorylation by MAPKs

Besides IKK/NF-κB signaling, MAPKKK-MAPKK-MAPK cascade is another crucial pathway involved in the TLR signaling (49, 50). As the MAPK cascade can be rapidly activated *via* either Myeloid differentiation primary response protein 88 (MyD88) or TIR-domain-containing adapter-inducing interferon-β (TRIF) (51, 52), two adaptors downstream of TLRs, several groups investigated the potential role of different MAPKs in NLRP3 inflammasome activation. Ghonime et al. studied early signaling events that occur before protein translation in human monocytes, and found that a short LPS priming time (5–30 min in duration) is sufficient for ATP-induced inflammasome activation (53). Moreover, by a small scale inhibitor screening, Ghonime et al. found that blockade of external signal regulated kinase1 (ERK1) significantly inhibits this rapid priming of NLRP3 in monocytes, but not immortalized mouse bone-marrow derived macrophage (BMDM). In contrast, transient knockdown of ERK2 cannot inhibit this priming process. Unfortunately, the direct target for this ERK1-dependent priming has not been determined in this study, although an indirect regulation of NLRP3 deubiquitination by ERK1 was suggested (53). In addition, regarding the differences in methodological, cell types, and species, priming mechanisms involving other MAPKs cannot be excluded.

To fully understand the phosphorylation profile of NLRP3, we previously performed a phospho-proteomic analysis by Flag-tag affinity purification plus liquid chromatography–mass spectrometry (LC-MS) (32). In the study, we have identified a series of phosphorylation sites in NLRP3 protein ranging from the N-terminal Pyrin domain to the C-terminal Leucine rich repeat (LRR) region. By mutagenesis evaluation in reconstituted NLRP3 inflammasome, we have identified that the phosphorylation of Ser194 in mouse NLRP3 (corresponding to Ser198 in human NLRP3) regulates the activation of NLRP3 inflammasome (32). Using the NLRP3-S194A knock-in mice, we demonstrated that NLRP3 S194A mutation significantly protects mice from LPS-induced endotoxemia and monosodium urate (MSU)-induced peritoneal inflammation. With NLRP3 phospho-Ser194 antibody, we found that this Ser194 phosphorylation is directly mediated by c-Jun N-terminal kinase 1 (JNK1), and occurs specifically in the priming process (32). Mechanistically, the phosphorylated NLRP3 is prone to form oligomers, according to the shift of NLRP3 into the high-molecular-weight fractions in gel filtration assay. These observations suggest that JNK1-mediated NLRP3 phosphorylation, as an essential priming event, is a prerequisite for inflammasome activation. In addition, inflammasome-related inflammatory responses have been proposed as the key pathogenic mechanism in endometriosis (54). Therefore, inhibition of inflammasome activation may alleviate the progression of endometriosis. The proof-of-concept studies have been performed by using JNK1 inhibitor Bentamapimod (also known as AS602801 and PGL5001). Palmer et al. showed that bentamapimod can reduce inflammatory cytokines in endometriotic lesions, and therefore repress the disease progression in an autologous rat endometriosis model (55). Consistently, Hussein et al. reported that bentamapimod can also reduce induced endometriosis in baboons, but without severe side effects (56). Together, these studies indicate JNK1 as a promising target for treating inflammasome-related disease.

Of interest, although the Ser194 phosphorylation of NLRP3 can be detected after priming, the level of this phosphorylation is relative low, suggesting that only a small portion of NLRP3 is phosphorylated by JNK1 (32). One possibility is that the phosphorylated NLRP3 may function as a “seed” to induce the conformational change of non-phosphorylated NLRP3, and to further nucleate the formation of the NLRP3 oligomers. A similar model of PRR oligomerization has been identified by two independent structure studies with regard to NLRC4 inflammasome (57, 58). Both of these studies showed that a single activated NAIP2 is sufficient for the initiation of NLRC4 polymerization *via* a domino-like reaction, in contrast to the assembly of apoptosome, in which each Apaf-1 needs to be activated by their ligands (59). Since NAIP2/NLRC4 and NLRP3 share similar oligomerization pattern (60), a detailed structure of NLRP3 would provide useful information to evaluate whether NLRP3 phosphorylation could also trigger structural reorganization and create the oligomerization surface like NLRC4.

Besides ERK and JNK, p38 is also a potent MAPK that modulates the inflammatory responses during infections (49). The role of p38 in the priming process has emerged recently. Fenini et al. reported that in primary human keratinocytes, two p38 MAPK isoforms, p38α and p38δ, are required for UVB-induced inflammasome activation and IL-1β secretion (61). In addition, Wang et al. showed that CDD-450, an inhibitor that selectively blocks p38α activation of downstream MAPKAPK2 (MK2), suppresses the activation of NLRP3 inflammasome without blockade of NLRP3 expression (62). Moreover, CDD-450 can attenuate neonatal-onset multisystem inflammatory disease (NOMID)-associated complications *in vivo*. As the NOMID-related NLRP3 mutation can trigger the assembly of inflammasome independent of the second signal, this observation indicates a potential role of p38-MK2 axis in the priming process. Considering p38 and JNK share some substrates such as myeloid cell leukemia 1 (Mcl-1) and activating transcription factor 2 (ATF2) (59, 63), it is likely that p38 may also be involved in NLRP3 phosphorylation in some specific cell types.

## Phosphorylation by p21-activated kinase (PAK)

Unlike the multiple phosphorylation sites identified in NLRP3 and ASC, only one report showed the phosphorylation of caspase-1. According to the finding by Basak et al. LPS derived from *Helicobacter pylori* can trigger the PI-3K/Rac1/p21-activated kinase 1 (PAK1) cascade, which in turn phosphorylates caspase-1 at Ser376 (36). As *H. pylori* LPS utilized in this study can directly induce the release of IL-1β, it is difficult to clarify whether this phosphorylation of caspase-1 occurs specifically in the priming step. However, the S376A mutation indeed blocks the activation of caspase-1. Of note, the Ser376 phosphorylation is located in the p10 subunit of caspase-1. Similarly, caspase-7, an executor caspase mediates apoptosis, also undergoes phosphorylation by PAK2 in its p11 subunit, whereas this Ser239 phosphorylation renders active caspase-7 incapable of binding substrate (64). Since both PAK1 and PAK2 can be activated by Rac1 (65), whether these phosphorylation modifications in two caspases orchestrate the balance between apoptosis and pyroptosis remains to be investigated.

Except for caspase-1, caspase 11 in mouse (corresponding to caspase 4/5 in human) is also involved in the host innate immunity, mainly by the formation of non-canonical inflammasome (66–69). So far, the intracellular LPS and lipid A have been proposed as the ligands for caspase-11 (69). The activated caspase-11 can cleave GSDMD and trigger the occurrence of pyroptosis (8). However, the caspase-11-induced secretion of IL-1β and IL-18 indeed requires the components of the NLRP3 inflammasome (68). Broz and colleagues indicated that caspase-11 can activate the canonical NLRP3 inflammasome by promoting K^+^ efflux (70). Considering the involvement of phosphorylation regulation of NLRP3 inflammasome, whether caspase-11 needs to be primed, or whether it is also modulated by phosphorylation is worthy of being studied.

## Phosphorylation in the activation step

In contrast to the regulation of priming step, the post-translational modification (PTM) of the activation step has been extensively investigated, profit by the abundance of readouts that can indicate the assembly of NLRP3 inflammasome. Since this assembly process involves a series of sequential molecular events, including the oligomerization of NLRP3, the recruitment of ASC and pro-caspase-1, as well as the cleavage of GSDMD (2), several phosphorylation modifications that regulates these events were clearly demonstrated.

## Phosphorylation in the pyrin domain

NLPR3, as the core initiator, has been evaluated via multiple strategies. Similar to the phosphoproteomic analysis that we performed, Stutz et al. utilized a murine cell line stably expressing the comparable level of NLRP3-FLAG to that of NLRP3 after priming (42). Among the sites identified, the phosphomimetic mutation of Ser5, which is located in the N-terminal PYD domain, completely abolished NLRP3 activation in response to nigericin. The PYD domain of NLRP3 is essential for the recruitment of ASC, and the NLRP3-ASC interaction is mediated largely by charge complementarity. Therefore the introduction of negative charges by Ser5 phosphorylation disturbs the interaction between NLRP3 and ASC. Although the kinase that phosphorylates the Ser5 of NLRP3 remains unclear, the phosphatase PP2A is involved in the dephosphorylation of Ser5. As aforementioned, PP2A, which is recruited to IKKα in response to both ATP and nigericin stimulation, regulates the association of ASC to NLRP3 (38). These observations indicate that okadaic acid (OKA), the PP2A inhibitor, may be a potent candidate for the treatment of NLRP3-related diseases. Of note, the N-terminal death domains, including both PYD and CARD domain, mediate the assembly of oligomeric signaling platforms, also referred to as signalosome (71). These homotypic interactions lead to the activation of the downstream signaling in several innate immunity pathways.

Besides NLRP3, RIG-I, and MDA5, two intracellular RNA sensors, also need to be dephosphorylated in their N terminal CARD domains during activation, respectively (72). However, this dephosphorylation process is mediated by PP1α and PP1γ, but not PP2A. Taken together, these studies indicate a possible unified mechanism for the activation of death domain-containing proteins, which relies on the on-off switch modulated by kinases and phosphatases.

## Phosphorylation in the NACHT domain

The NACHT domain of NLRP3 is closely related to the inherited autoimmune diseases such as CAPS (25–27). A series of mutations have been identified in this domain, leading to the autoactivation of NLRP3 independent of signal 2 stimulation. Consistently, ATP hydrolysis by the NACHT domain has been reported to regulate NLRP3 self-oligomerization (73). Although none of the phosphorylation site identified in this domain was shown to be essential for the regulation of NLRP3 activation, the PKA-induced Ser295 phosphorylation (corresponding to Ser291 in mouse NLRP3) significantly blocks nigericin-induced inflammasome activation (39). This Ser295 phosphorylation can induced either by chemicals such as forskolin, or endogenous compounds like bile acids, both of which trigger the cAMP-PKA signaling (40). Of interesting, the inhibitory effect of cAMP on NLRP3 inflammasome activation has already been proposed in an earlier study, which indicates that cAMP can directly bind to NLRP3, leading to the inhibition of inflammasome assembly (74). Albeit the differences in the indicated mechanisms, all these studies demonstrated that both the phosphorylation of Ser295 and cAMP treatment, showed marginal effect on the CAPS-related NLRP3 mutants. This observation further confirmed that targeting the priming step, as well as the interaction between ASC and NLRP3, might be more potent strategies for treating CAPS than targeting the NACHT domain of NLRP3.

In contrast to the inhibitory effect of Ser295 phosphorylation on NLRP3 inflammasome, Zhang et al. showed that protein kinase D (PKD)-induced Ser295 phosphorylation at Golgi is also required for NLRP3 activation, *via* facilitating the release of NLRP3 from mitochondria-associated membrane (MAM) (41). Moreover, the inhibition of PKD prevents inflammasome activation in peripheral blood mononuclear cells (PBMCs) derived from CAPS patients. One possible explanation for this discrepancy is that the Ser295 phosphorylation of NLRP3 exhibit dual functions with regard to the inflammasome activation. Therefore, a sequential phosphorylation-dephosphoryation process is required for the activation of NLRP3 inflammasome. Similar mechanism has also been proposed in the activation of Pyrin inflammasome. As reported by Gao et al. the phosphorylation of Ser205 and Ser241 in mouse Pyrin resulted in inhibitory binding of cellular 14-3-3 proteins, while the signal-induced dephosphorylation of Pyrin at these two sites further licenses the activation of Pyrin inflammasome (75). Taken together, the phosphorylation of Ser295 may be an important regulation for NLRP3 activation, while the detailed function of this phosphorylation still requires further clarification.

## Phosphorylation in the LRR domain

Tyr861 phosphorylation, as the first reported phosphorylation modification of NLRP3, negatively regulates the activation of NLRP3 (76). Spalinger et al. showed that Protein tyrosine phosphatase, non-receptor type 22 (PTPN22) directly interacts with NLRP3 and thus dephosphorylates NLRP3. Intriguingly, the Y861C mutation of NLRP3 has been identified in patient with chronic infantile neurologic cutaneous and articular syndrome (CINCA), consistent with the result that the Y861F mutation of NLRP3 indeed exhibits an elevated level of inflammasome activation, compared with wild-type NLRP3. Moreover, PTPN22 affects NLRP3 activation only in the lamina propria, but not in epithelial cells, due to the restricted *Ptpn22* mRNA expression. As the pathogenesis of CAPS including CINCA is not limited in the lamina propria (25–27), whether this PTPN22-mediated NLRP3 phosphorylation can suppress CAPS and other NLRP3-driven disorders is still unclear. In addition, unlike other CAPS-associated NLRP3 mutation, Y861F alone is not sufficient to trigger the inflammasome activation independent of signal 2, suggesting that this Tyr861 phosphorylation may regulate the NLRP3 activation specifically in some circumstances such as colitis. Since the kinase that directly phosphorylates Tyr861 remains to be identified, further studies using phospho-Tyr861 antibody and gene-editing mice would provide more information for understanding the role of PTPN22 on NLRP3 activation.

## Phosphorylation in the CARD domain

In addition to NLRP3, ASC phosphorylation may also occur during the activation step. Hara et al. suggested that Syk/JNK1/JNK2-mediated ASC phosphorylation plays a role in the pyroptosome formation of NLRP3 and AIM2, but not NLRC4, inflammasome (37). Inhibition of Syk or JNK abolished the formation of ASC specks, while does not affect the interaction of ASC with NLRP3. By mutagenesis assay, Hara et al. identified the Tyr144 of ASC as a possible phosphorylation site, which is critical for speck formation. Of note, the role of Syk and JNK proposed in this study is contradictory to previous findings. Gross et al. reported that Syk is not required for ATP or nigericin-induced NLRP3 inflammasome activation, but required for anti-fungal host defenses (77). Moreover, JNK2 showed an inhibitory effect on NLRP3 inflammasome activation (78). JNK1, as a serine/threonine-specific protein kinase, is not likely the kinase that mediates the Tyr144 phosphorylation. In addition, the role of ASC in the activation of NLRC4 inflammasome is still under debate, as the N terminal CARD domain of NLRC4 is sufficient for the recruitment of NLRC4. According to the *in vitro* reconstitution assay, NLRC4 can directly activate caspase-1 independent of ASC expression (79). However, ASC oligomerization has indeed been observed in primary BMDMs triggered with NLRC4 stimuli. Furthermore, knockout of ASC significantly disrupts the NLRC4 inflammasome activation in BMDMs (80). In contrast to these observations, Hara et al. showed that NLRC4 inflammasome activation is not regulated by Syk/JNK1/JNK2-mediated ASC phosphorylation, indicating the binding pattern of ASC and NLRC4 may be different from that of ASC and other PYD-containing PRRs.

Indeed, Chung et al. reported that Pyk2, downstream kinase of Syk, directly phosphorylates ASC at Tyr146 (44). Upon nigericin treatment, Pyk2 is activated by phosphorylation at Tyr402. The phosphorylated Pyk2 co-localizes with ASC, facilitating the formation of ASC oligomerization by Tyr146 phosphorylation. Moreover, Pyk2 inhibitor PF-431396 downregulates NLRP3- and AIM2-induced IL-1β secretion and pyroptosis (44). In addition, the clinical-trial-tested Pyk2/FAK dual inhibitor PF-562271 mitigates monosodium urate-mediated peritonitis *in vivo*. Syk signaling can be activated by integrins (81). Consistently, Integrin α5β1 has been reported to activate inflammasome by direct interaction with a bacterial surface protein (82), suggesting that the Syk signaling plays a critical role in the regulation of inflammasome activation. Whether other tyrosine phosphorylations such NLRP3 Tyr861 may be regulated by Syk remains to be further clarified.

Besides Pyk2, Bruton's tyrosine kinase (BTK) can also be triggered by Syk (83). Ito et al. performed a screening of pharmacological signal inhibitors, and found that BTK is an essential regulator of the NLRP3 inflammasome (84). BTK inhibition substantially reduced ASC redistribution into the Triton X-insoluble fraction in macrophages in which NLRP3 inflammasome is activated. Moreover, BTK-induced redistribution of ASC can be severely reduced in the presence of the BTK inhibitor. In addition, a recent study indicates that the approved BTK inhibitor ibrutinib blocks the secretion of IL-1β in cells derived from patients with Muckle-Wells syndrome (85). Together with the fact that BTK is a tyrosine kinase, BTK kinase activity is also likely to be required for ASC phosphorylation. Further studies using a specific ASC phosphorylation antibody would promote our understanding of the role of this PTM regulation in ASC oligomerization.

## Concluding remarks

In the past decade, extensive efforts have been put into the exploring of the regulation mechanisms of NLRP3 inflammasome. Although many interacting proteins and post-transcriptional modifications have been suggested to be essential for either the priming of NLRP3 or its activation, the detailed mechanisms of NLRP3 regulation are still needed to be elucidated. Of note, many studies that focus on the PTM regulation of NLRP3 highly rely on the *in vitro* system, therefore the contradictories may be caused due to the different methodologies utilized in either the generation of stable cell lines, or other function assays. Future studies performed *in vivo* would provide a more convincing clue for understanding the regulation of NLRP3 inflammasome. Given the fact that the aberrant NLRP3 inflammasome activation is involved in the pathology of various inflammatory disorders, including CAPS, gout, type 2 diabetes, obesity, as well as neurological diseases, what is the unified mechanism that modulates the NLRP3 activation in response to a wide spectrum of stimuli need to be further investigated. Finally, clinical investigation of compounds such as MCC950 and the ketone metabolite β-hydroxybutyrate (BHB), which specifically targets NLRP3, may lead to the development of therapies against NLRP3-related diseases. Investigation into the mechanism of NLRP3 inflammasome activation will aid the development of NLRP3-targeted compounds for treatment of NLRP3-driven diseases.

## Author contributions

NS drafted the manuscript. TL supervised and edited the manuscript.

### Conflict of interest statement

The authors declare that the research was conducted in the absence of any commercial or financial relationships that could be construed as a potential conflict of interest.
